# Anticancer Properties and Phenolic Contents of Sequentially Prepared Extracts from Different Parts of Selected Medicinal Plants Indigenous to Malaysia

**DOI:** 10.3390/molecules17055745

**Published:** 2012-05-14

**Authors:** Maznah Ismail, Bagalkotkar Gururaj, Iqbal Shahid, Hadiza Altine Adamu

**Affiliations:** 1 Laboratory of Molecular BioMedicine, Insitute of Bioscience, University Putra Malaysia, 43400 UPM, Serdang, Selangor, Malaysia; Email: ranashahid313@gmail.com (S.I.); 2 Department of Nutrition and Dietetics, Faculty of Medicine and Health Sciences, University Putra Malaysia, 43400 UPM, Serdang, Selangor, Malaysia; 3 Department of Chemistry, University of Sargodha, Sargodha 40100, Pakistan

**Keywords:** anticancer activity, MTS assay, total phenolic content, medicinal plants

## Abstract

Different parts of four edible medicinal plants (*Casearia capitellata*, *Baccaurea motleyana*, *Phyllanthus pulcher* and *Strobilanthus crispus*), indigenous to Malaysia, were extracted in different solvents, sequentially. The obtained 28 extracts were evaluated for their *in vitro* anticancer properties, using the MTS assay, on four human cancer cell lines: colon (HT-29), breast (MCF-7), prostate (DU-145) and lung (H460) cancers. The best anticancer activity was observed for the ethyl acetate (EA) extract of *Casearia capitellata* leaves on MCF-7 cell lines with IC_50_ 2.0 μg/mL and its methanolic (MeOH) extract showed an outstanding activity against lung cancer cell lines. Dichloromethane (DCM) extract of *Phyllanthus pulcher* aerial parts showed the highest anticancer activity against DU-145 cell lines, while significant activity was exhibited by DCM extract of *Phyllanthus pulcher* roots on colon cancer cell lines with IC_50_ value of 8.1 μg/mL. Total phenolic content (TPC) ranged over 1–40 mg gallic acid equivalents (GAE)/g. For all the samples, highest yields of phenolics were obtained for MeOH extracts. Among all the extracts analyzed, the MeOH extracts of *Strobilanthus crispus* leaves exhibited the highest TPC than other samples (*p* < 0.05). This study shows that the nature of phenol determines its anticaner activity and not the number of phenols present.

## 1. Introduction

Plant based drugs have a long history in both traditional and modern societies as herbal remedies or crude drugs, or as purified compounds approved by the Food and Drug Administration and similar regulatory agencies [1,2,3]. Drug discovery from plants still provides important new drugs, many of which are approved or have undergone trials for clinical uses against cancer, malaria, Alzheimer’s disease, HIV/AIDS, pulmonary pathologies and other diseases [3,4]. However, plant-based drugs present many challenges, including legal and logistic difficulties involved in the procurement of plant materials [5,6], the lengthy and costly processes of bioassay-guided fractionation and compound isolation [1,2], and the elimination or scaling down of natural product research programs at pharmaceutical companies and governmental agencies [3,7].

Investigations about natural products have recently regained prominence with the rapidly increasing understanding of their biological significance and increasing recognition of the origin and function of their structural diversity.

Cancer is a leading cause of death worldwide. Breast and prostate cancer are two of the most common malignancies and contribute significantly to the societal and economic burden of cancer. About 10 million new cases are diagnosed and over 6 million deaths occur worldwide annually (excluding non-melanoma skin cancers) [8]. Important progress has been made in cancer chemotherapy, a considerable portion of which may surely be attributed to plant-derived drugs [4]. Epidemiological and experimental studies reveal a negative correlation among the consumption of fruits or vegetables and the risks for chronic diseases, including cancer [9,10,11,12]. The physiological functions of fruits and vegetables may be partly attributed to their abundance in phenolic compounds [11,13]. The American Cancer Society recommends abundant foods from plant-based dietary resources and to eat five or more servings of fruits and vegetables each day, in addition to other foods from plant sources, such as whole grains and beans, which are recommended to be taken several times a day. Green and dark yellow vegetables, beans, soybean products and cruciferous vegetables, such as broccoli, brussels sprouts and cabbage are expected to reduce the risk of cancer.

The present paper deals with the screening of the following medicinal plants: *Phyllanthus pulcher* (Euphorbiaceae), *Casearia capitellata* (Flacourtiaceae), *Strobilanthes crispus* (Acanthaceae) and *Baccaurea motleyana* (Phyllanthaceae) for their TPC and anticancer potential. Plants selected for this study were chosen because of their use in local traditional cuisine. Information on the traditional culinary purposes of these plants was collected through a structured literature review. 

The study shows *P*. *pulcher* from genus *Phyllanthus* is found throughout South East Asia and is locally (in Malaysia) known as Naga Buana (Dragon of the World). The plant is used extensively in Malay traditional medicines: as a decoction for stomach ache, the leaves to treat tooth ache, poultices are applied to the nose for ulceration, to the skins to treat boils, and to the abdomen of children with kidney problems [14]. *C. capitellata* from the family of Flacourtiaceae is a lowland rain forest treeendemic to Malaysia. *Casearia* species are traditionally used in Malaysia as antiseptic, cicatrizant and topical anasthetic agent [14]. They have antiulcer [15], antiinflammatory [16], antihyperglycaemic [17], and hypolipidaemic [18] activities, as well as snake and bee venoms neutralization capacity [19,20]. *Strobilanthes* is a genus of about 250 species of flowering plants in the family Acanthaceae, mostly native to tropical Asia, but with a few species extending north into temperate regions of Asia. Traditionally, the leaves of *S. crispus* were boiled with water and the filtrates were used in traditional medicine in Malaysia and Indonesia as antidiabetic, diuretic, antilytic, and laxative. *S. crispus* has been proven to be possessing high antioxidant and antihyperglycemic properties [21,22]. *Baccaurea motleyana* (Family: Phyllanthaceae) is a species of fruit tree, which grows wild in parts of Southeast Asia and is cultivated for its fruit in Thailand and Peninsular Malaysia. The fruits are approximately 2 to 5 centimeters long and about too wide and grow in strands. Each fruit has velvety pinkish, yellow, or brown skin, which wrinkles at ripening and is filled with whitish pulp containing 3 to 5 seeds. The pulp is sweet to acidic in taste. The fruits are eaten fresh or processed into beverages and wine. The tree is also used for shade and low-quality wood [14].

A survey of the literature revealed that no studies on the anticancer potential of extracts from these plants have been undertaken on four human cancer cell lines: breast cancer cell line (MCF-7), prostate cancer cell line (DU-145), colon cancer cell line (HT-29) and lung cancer cell line (H460). It is known that different cell lines might exhibit different sensitivities towards an anticancer compound, so the use of more than one cell lines was therefore considered necessary in the evaluation of anticancer effects. Bearing this in mind, four human cell lines of different histological origin were used in the present study. The study is aimed to evaluate anticancer potential of sequentially prepared extracts from different parts of these plants on above said cell lines. The aim of this study was so to investigate the anticancer activity of these extracts against a panel of tumor cell lines. The TPC from these extracts was also determined. The findings of the study will be helpful in understanding anticancer and antioxidant potential of these plants and significance for the development of new anticancer drugs.

## 2. Results and Discussion

### 2.1. Extraction and Determination of Total Phenolic Content (TPC)

**Figure 1:**
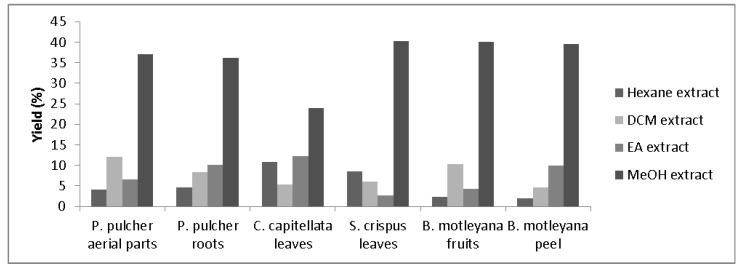
Yield of the extracts obtained from different parts of plants employing sequential extraction scheme (DCM = Dicholoromethane, EA = Ethyl acetate, MeOH = methanol).

The yield of extracts, from different parts of all plants prepared sequentially in different solvents, is shown in Figure 1. The yield varied over a wide range among the solvents and plants, *i.e.*, 2.0 to 40%. However, the highest yield was obtained for methanolic extracts. It is in accordance with earlier observations [23], reporting that the highest amount of antioxidative compounds is extractable in methanol. 

**Figure 2:**
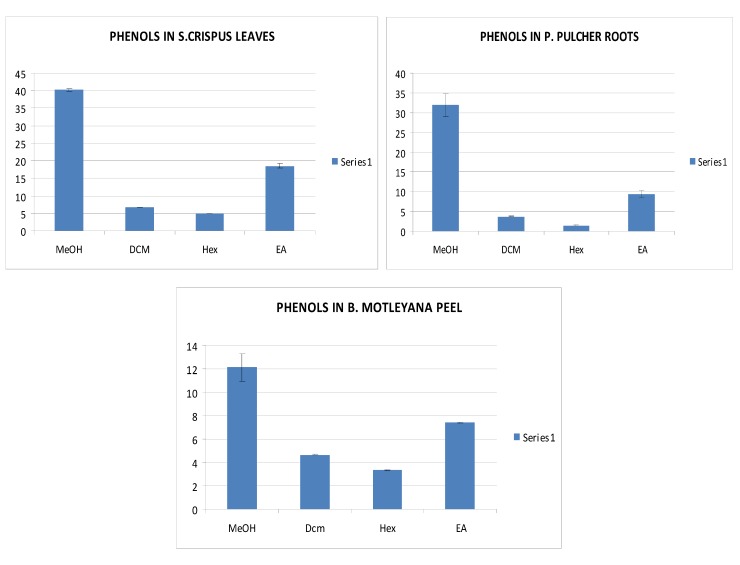
Phenolic content in *S. crispus* leaves, *P. pulcher* roots and *B. motleyana* peels.

Total phenolic contents were determined spectrophotometrically using Folin-Ciocalteu reagent and results are presented in Table 1. It should be mentioned that these results are an estimation of TPC in chemical equivalents (gallic acid), since different phenolic compounds contribute differently to total absorbance when using the Folin–Ciocalteau reagent for total phenolic content determination. The amounts of TPC were found to vary over a wide range (1 to 40 mg/g of extract) among the extracts (Table 1). Among all the tested plant extracts, MeOH extract of *S. crispus* leaves contained the highest amount of phenolics (40 mg/g) followed by *P. pulcher* roots (32 mg/g), *C. apitellata* (30.4 mg/g), *B. motleyana* fruit (21 mg/g), *S. crispus* flowers (17 mg/g), *P. pulcher* aerial parts (15 mg/g), whereas the lowest level was found in *B. motleyana* peel (12 mg/g) (Figure 2). Phenols were found in highest concentrations in *S. crispus* leaves (40 mg/g) as compared to *S. crispus* flowers (17 mg/g), while *P. pulcher* roots (32 mg/g) contained more phenols than *P. pulcher* aerial parts (15 mg/g). Whereas *B. motleyana* fruits (21 mg/g) contained more phenols in comparison to *B. motleyana* peel (12 mg/g).

**Table 1:** Determination of total phenolics in extracts of *P. pulcher*, *C. capitellata*, *S. crispus* and *B. motleyana*.

Extracts	Total phenolic content (mg gallic acid Eq/g extract)						
	*P. pulcher* Aerial parts	*P. pulcher* Roots	*C. capitellata* Leaves	*S. crispus* Flowers	*S. crispus* Leaves	*B. motleyana* Fruits	*B. motleyana* Peel
Hexane	3.4 ± 0.3	1.4 ± 0.6	1.9 ± 0.1	5.3 ± 0.5	4.9 ± 0.4	2.1 ± 0.1	3.3 ± 0.1
DCM	9.7 ± 0.3	3.7 ± 0.2	5.0 ± 0.8	7.3 ± 0.2	6.7 ± 0.1	4.6 ± 0.3	4.7 ± 0.3
EA	11.2 ± 0.3	9.4 ± 0.2	11.8 ± 0.6	10.4 ± 0.2	18.6 ± 0.9	6.4 ± 0.9	7.4 ± 0.6
MeOH	15.4 ± 0.4	32.0 ± 2.9	30.0 ± 0.3	17.0 ± 0.5	40.2 ± 0.7	21.0 ± 0.6	12.0 ± 1.9

Results are expressed as mean (n = 3) ± SD (n = 3). Total phenolics were significantly different in medicinal plants as well as among their extracts (*p* < 0.05).

The variation among TPC may be assumed due to the presence of different types of phenols and other constituents in different parts/plants; selectively extractable depending upon the extraction medium, *i.e.*, solvent employed. Having used several solvents, it was found that the best yields of phenolic compounds, for all the samples, were obtained in high polar medium, *i.e.*, MeOH extracts. This is in agreement with a recent study on *Pistacia vera*, where it was found that the yield in total phenols depends on the method and the choice of solvent, and that the highest amount was obtained in highly polar extracts (34.7 mg of tannic acid equivalent/g of plant powder) [24].

The variations in amounts of phenolics in these plant extracts could be partly due to the differences in growing conditions. Under field conditions, the phenolic compositions of plant tissues are reported to be varying considerably with seasonal, genetic, and agronomic factors [25]. In addition, a large variability at different stages of maturation and growing conditions such as temperature and rainfall is known to affect the TPC [26].

### 2.2. Anticancer Activity

All the four cell lines, used in this assay, were capable of attachment to form a homogeneous monolayer on plastic substratum of culture wells, which is ideal for MTS assay. The MTS test is a simple bioassay used for the primary screening of crude plant extracts and isolated compounds. For each cell line, there was a linear relationship between cell number and absorbance; measured at 550 nm in both control and drug-treated wells. After 72 h of treatment, the anticancer activity of the plant extracts was determined. The anticancer activity (IC_50_ values) of plant extracts on the growth of MCF-7, HT-29, DU-145 and H460 cell lines, measured by MTS assay, are given in Table 2.

**Table 2:** Anticancer activity of extracts from different parts of selected plants prepared in different solvents.

Extracts	IC_50_ (μg/mL)			
	MCF-7 cells	DU-145 cells	H460 cells	HT-29 cells
*P. pulcher* aerial parts				
Hexane extract	NA	NA	NA	NA
DCM extract	8.0 ± 0.1	13.4 ± 0.4	50.0 ± 1.5	22.0 ± 0.8
EA extract	NA	NA	NA	NA
MeOH extract	NA	NA	NA	NA
*P. pulcher* roots				
Hexane extract	NA	NA	NA	NA
DCM extract	9.8 ± 1.0	16.7 ± 1.3	26.0 ± 2.0	8.1 ± 0.5
EA extract	18.6 ± 3.4	20.8 ± 2.6	42.0 ± 4.1	67.3 ± 1.4
MeOH extract	NA	NA	NA	NA
*C. capitellata* leaves				
Hexane extract	NA	NA	NA	NA
DCM extract	11.4 ± 2.6	17.0 ± 2.0	24.7 ± 1.3	42.0 ± 1.2
EA extract	2.0 ± 0.3	14.5 ± 0.5	23.4 ± 2.3	72.3 ± 0.6
MeOH extract	8.6 ± 0.1	14.4 ± 1.7	12.7 ± 2.1	25.6 ± 1.3
*S. crispus* leaves				
Hexane extract	NT	NT	NT	NA
DCM extract	NT	NT	NT	NA
EA extract	NT	NT	NT	70.2 ± 1.4
MeOH extract	NT	NT	NT	59.0 ± 0.8
*S. crispus* flowers				
Hexane extract	NT	NT	NT	NA
DCM extract	NT	NT	NT	90.3 ± 1.1
EA extract	NT	NT	NT	42.0 ± 1.8
MeOH extract	NT	NT	NT	NA
*B. motleyana* fruits				
Hexane extract	NT	NT	NT	51.0 ± 3.1
DCM extract	NT	NT	NT	82.4 ± 2.4
EA extract	NT	NT	NT	NA
MeOH extract	NT	NT	NT	NA
*B. motleyana* peel				
Hexane extract	NT	NT	NT	43.6 ± 0.3
DCM extract	NT	NT	NT	75.0 ± 1.2
EA extract	NT	NT	NT	NA
MeOH extract	NT	NT	NT	NA

Results are expressed as mean (n = 3) ± SD (n = 3); NA = no activity; NT = not tested.

**Figure 3:**
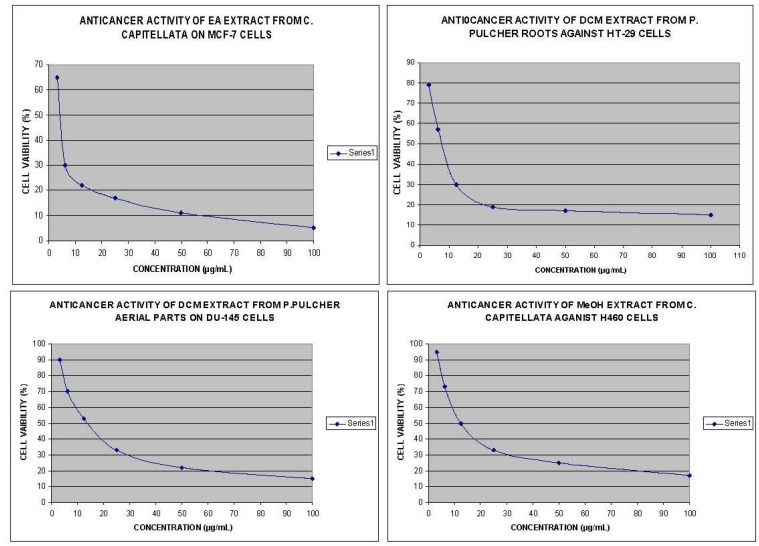
Dose response curves of extracts from *C. capitellata* and *P. pulcher* against four human cancer cell lines.

Cell type anticancer specificity is observed in some plant extracts. This specificity of plant extracts is likely to be due to the presence of different classes of compounds in the extract, as it has been documented in the case of known classes of compounds [27]. The best anticancer activity was exhibited by EA extract of *C. capitellata* that showed IC_50_ value of 2 μg/mL on MCF-7 cell line (Figure 3). Furthermore, MeOH extract of this plant showed significant activity on all the tested human cancer cell lines with IC_50_ values ranging from 8.6–25.6 μg/mL. Methanolic extracts of *C. capitellata* exhibited the best anticancer activity against H460 cell lines, with an IC_50_ value of 12.7 μg/mL (Figure 3). Generally, all the extracts (except hexane extract) from *C. capitellata* showed good anticancer activity against all the tested cell lines. DCM extract of *P. pulcher* roots showed the best anticancer activity on HT-29 cell line with IC_50_ value of 8.1 μg/mL, while significant activity was exerted by DCM extract of *P. pulcher* aerial parts on DU-145 cell line with IC_50_ value of 13.4 μg/mL (Figure 3). Furthermore, DCM extract of this plant showed significant activity on all the tested human cancer cell lines with IC_50_ values ranging over 8.0-50.0 μg/mL. Whereas the extracts of *P. pulcher* aerial parts, prepared in other solvents, at the highest concentration of 100 μg/mL failed to exhibit any anticancer effects. In general, DCM extract from aerial parts of *P. pulcher* was more active than DCM extract from roots of *P. pulcher* against the tested cancer cell lines, being selective towards MCF-7. Flower extracts from *S. crispus* were observed to be more active than leaves extracts of *S. crispus* against HT-29 cell lines, whereas *B. motleyana* peel extracts were more potent than *B. motleyana* fruit extracts on HT-29 cell lines.

Particularly DCM extract of *P. pulcher* aerial parts showed a selective anticancer activity on breast cancer while significant activity was exerted by EA extract of *C. capitellata* on lung cancer (Figure 3). Whereas, DCM extract of *P. pulcher* roots showed the highest anticancer activity against colon cancer cell lines. Several studies have evaluated the relations between anticancer activity of plant extracts and their phenolic content. Previous studies suggest that a correlation exists between the structural oxidation state and the position, number, and nature of substituents of the polyphenolic compounds and their anticancer effects [28]. The effect of phenolics on the cell cycle could probably contribute to the tumor cell killing [29]. Phenolic compounds are reported to be exerting a direct anticancer action, evident at low concentrations even, comparable with those found in biological fluids after ingestion of foods rich in phenolic compounds. Furthermore, the direct interaction with the aryl hydrocarbon receptor, the nitric oxide synthase inhibition and their pro-apoptotic effect provides some insights into their biological modes of action [30].

In this study, however, the findings did not show any relationship between anticancer activity and phenol composition. Extracts with higher anticancer activity didn’t necessarily show a high phenol composition. For example, *C. capitellata*, which showed a medium phenolic composition, exhibited a high anticancer activity whereas *S. crispus* leaves which contained the highest phenolic compounds failed to show any anticancer activity. The relatively high anticancer activity of the extracts containing low phenolic composition suggests that the nature of phenols is determinant for these activities rather than their amounts. 

## 3. Experimental

### 3.1. Materials

The different botanical taxa studied in this work are shown in Table 3. Information about their common names, parts of the plants used in this study and their traditional uses is also incorporated. The collected plants were authenticated and specimens were deposited at IBS herbarium. Hexane, chloroform, ethyl acetate, and methanol were used for extraction and were purchased from Fisher Scientific *,* UK. All other chemicals were procured from Sigma Chemical Co. (USA). 

**Table 3:** Scientific and common names of plants used in this study; parts and traditional uses.

Scientific name (family)	Common name	Plant part used	Traditional uses
*Phyllanthus pulcher* (Euphorbiaceae)	Naga Buana	Leaves, stems and roots	Ulcer and kidney problems
*Casearia capitellata* (Flacourtiaceae)	Simmilit matangi	Leaves	Antiseptic and anesthetic agent
*Strobilanthes crispus* (Acanthaceae)	Pecah kac or Jin batu	Leaves and flowers	Antidiabetic, diuretic and antioxidant
*Baccaurea motleyana* (Phyllanthaceae)	Rambai	Fruits and peel	For stomachache and sore eyes

Breast cancer (MCF-7), prostate cancer (DU-145), colon cancer (HT-29) and lung cancer (H460) cell lines were obtained from American Type Culture Collection (ATCC, Manassas, VA, USA). Sample absorbance was recorded by a Shimadzu UV-1700 pharma spectrophotometer at 765 nm.

### 3.2. Extraction Procedure

The plant material was subjected to dryness at room temperature, cut into small pieces and was then ground to a fine powder using an electric mill. The cold extraction method using organic solvents was employed to obtain crude extracts from all the plants. Dried powdered plant materials were sequentially extracted with hexane, dichloromethane (DCM), ethyl acetate (EA) and methanol (MeOH). The extraction process was conducted thrice to ensure the complete extraction of required materials and the combined filtrate, for individual sample, was concentrated using a rotary evaporator. 

### 3.3. Determination of Total Phenolic Content (TPC)

Total phenolic contents (TPC) were determined using Folin-Ciocalteu reagent as described earlier [23] with slight modifications. About 0.3 mL of extract (taken in triplicate), 1.5 mL of diluted Folin-Ciocalteu reagent (1:10) and 1.2 mL of sodium carbonate (7.5 g/100 mL) were added in a test tube. The resulting mixture was then vortexed for 10 seconds and stored in dark for 30 min. The absorbance of the resulting solution was then recorded spectrophotometrically at 765 nm. Water served as the blank. Gallic acid was used as calibration standard and results were expressed as mg gallic acid equivalents (GAE)/g dry weight (DW) of extract.

### 3.4. Cell Culture

Cells were grown in RPMI-1640 culture medium supplemented with 10% heat-inactivated foetal bovine serum, 100 IU/mL of penicillin and 100 μg/mL of streptomycin. Subconfluent cells were trypsinised using trypsin-EDTA after every 3–4 days and cells were placed in 3 mL of medium and syringed to obtain a suspension of single cells. The cell lines were maintained at 37 °C in 5% CO_2_ atmosphere with 95% humidity. The optimal plating density of cell lines was determined to be 1 × 10^3^ to ensure exponential growth throughout the experimental period and to ensure a linear relationship between absorbance at 550 nm and cell number when analyzed by the 3-(4,5-dimethylthiazol-2-yl)-5-(3-carboxymethoxyphenyl)-2-(4-sulfophenyl)-2H-tetrazolium (MTS) cytotoxicity assay to determine the anticancer effects of all plant extracts against a panel of cancer cell lines. The MTS cytotoxic assay has been considered a valuable tool in studying cytotoxicity induced by chemical agents, for many years. It uses a tetrazolium salt, which is reduced to a coloured formazan product by mitochondrial dehydrogenases in living cells. It is frequently used and is reported to be suitable for *in vitro* drug screening tests [31]. Cells were counted using a Neubauer haemocytometer and plated in 96-well flat bottom tissue culture plates at initial seeding densities between 1 × 10^5^ cells per well and in a volume of 180 µL of culture media. The plates were incubated at 37 °C (5% CO_2_ and 95% air) overnight to allow attachment onto the wells, before the addition of plant extracts in replicates of 4 wells for each concentration to give final concentrations in the range of 0.1–100 µg/mL. Following incubation at 37 °C in an atmosphere of 5% CO_2_ and 95% air for 72 h, 50 µL of MTS (2 mg/mL in PBS) were added into each well containing the cells and medium above (blank). The plates were re-incubated for 1–4 h to allow metabolism of MTS by cellular mitochondrial dehydrogenases. The viable cells converted soluble yellow MTS into insoluble purple formazan. Following the incubation period, the medium containing MTS was aspirated from each well and 100 µL of DMSO was added to dissolve formazan in order to read the absorbance of soluble formazan at 550 nm, corresponding to number of living cells. The absorbance was determined at 550 nm using a 96-well microplate reader. The percentage of cell viability was calculated using the formula:


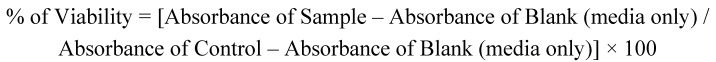


Dose-response curves (% of viability Vs concentration) were constructed to obtain 50% inhibitory concentrations (IC_50_).

### 3.5. Statistical Analysis

All the experiments were conducted in triplicate and statistical analyses was done according to SPSS 12.0. Data were expressed as means ± S.D. Statistical analysis was performed by one-way analysis of variance, followed by Duncan’s multiple range tests to determine the significance of differences among the means and multiple comparisons of unpaired data. Differences were considered significant at *p* < 0.05. The inhibitory concentration 50% (IC_50_) was calculated from the dose–response curves (statistical programme) obtained by plotting the percentage of inhibition *versus* the concentrations.

## 4. Conclusions

In conclusion, this work reveals that selected Malaysian flora species, including *C. capitellaat*, *P. pulcher* and *S. crispus*, can be an interesting source of anticancer compounds. Further studies identifying and isolating the key phenolic compounds may allow for a potential biomedical application in the therapy of cancer diseases.
